# Reviewing the use of the Brief Religious Coping Scale (Brief RCOPE) across diverse cultures and populations

**DOI:** 10.1007/s10943-024-02119-z

**Published:** 2024-09-06

**Authors:** Nicola Saunders, Zoe Stephenson

**Affiliations:** University of Birmingham, Birmingham, UK

**Keywords:** Brief RCOPE, Coping, Psychometric measure, Life stressors, Reliability

## Abstract

To discern the religious coping methods which individuals employ when confronting life stressors, Pargament devised the Religious Coping Scale (RCOPE) in 1997. Subsequently, in 1998, Pargament et al. formulated an abbreviated iteration, the Brief RCOPE, intended for both research and practical application. The Brief RCOPE has been found to be a reliable and valid measure, however much of the research looking into the psychometric properties of this measure has been conducted in the United States. The aim of the current review was to draw together findings from studies which have investigated the reliability and/or validity of the measure amongst populations outside of the United States. A narrative approach was adopted, involving searches of academic databases using keywords and the application of specific inclusion criteria. It was found that the reliability of the measure has been demonstrated across a number of countries, and across a range of different religions. The measure has also demonstrated good convergent, construct and concurrent validity in diverse cultures. The findings from this review suggest that the Brief RCOPE can be used in a range of diverse religions and cultures. However, more research is needed to ascertain the accuracy of the measure with other religions and cultures and with those in minority groups.

## Introduction

According to coping theory (Lazarus & Folkman, 1984), individuals address life stressors proactively by seeking meaning and significance in their lives. Many individuals employ a method of turning to their religion when confronted with challenging life circumstances (Rambo, 1993), and ongoing research has uncovered significant links between religion and mental health (Abu-Raiya & Pargament, 2014). For instance, Bradshaw et al. (2010) discovered that a perceived secure attachment to God is linked with enhanced abilities to cope with distress, while Ellison et al. (2014) reported that individuals exhibiting higher levels of religious commitment experience greater mental health benefits.

Additionally, Koenig et al. (2015) discovered that religious interventions seem to be particularly efficacious in alleviating distress among individuals with higher levels of religiosity. These findings have been replicated across various religious traditions, encompassing Islam, Judaism, Hinduism, and Buddhism (Abu-Raiya & Pargament, 2014).

Initial investigations into religious coping concentrated on assessing the impact of religion by gauging correlations between intensity of religious convictions or the frequency of church attendance, and distress levels (Ano & Vasconcelles, 2005). However, this perspective is deemed an oversimplification of the concept of religion and its multifaceted role in individuals’ coping mechanisms for navigating stressful life events (Pargament et al., 1998). To develop our understanding of the role of religion as a coping strategy, it is important to assess how an individual uses religion to understand and to deal with stressors (Pargament et al., 2000, 2013).

A critique directed against prior methodologies in the measurement of religious coping is their failure to attain a comprehensive understanding of the nuanced ways in which religiosity and prayer serve as mechanisms for coping. For example, merely quantifying the frequency of prayer, overlooks the underlying motivations behind prayer and its functional role (Pargament et al., 2011). Furthering our understanding of religious coping was the basis for Pargament’s (1997) decision to develop a measure of religious coping; attempting to provide a theoretical framework from which to understand religious issues and to incorporate them into assessment and intervention (Pargament et al., 2000).

### Theory of Religious Coping

Pargament (1997) defined religious coping as “efforts to understand and deal with life stressors in ways related to the sacred” (Pargament et al., 2011, p. 52). In his theory of religious coping, he made a number of assumptions as to how religion is used to understand and cope with negative life events (Pargament & Raiya, 2007): (1) Religious coping is multi-functional, including searching for meaning, intimacy with others, comfort, control, and life transformation; (2) Religious coping involves individuals employing emotions, relationships, cognition and behaviour; (3) Religious coping changes over time and situation, in that ways in which people use religion to cope can vary depending on the context of the life event; (4) Religious coping differs from other coping strategies as it involves the addition of the ‘sacred’; (5) Religious coping can include both adaptive and maladaptive strategies (Mohammadzadeh & Najafi, 2016).

Pargament’s (1997) theory of religious coping was used to guide the development of a measure of religious coping, called the Religious Coping Scale (RCOPE), the aim of which was to identify the religious coping methods that people employ when dealing with major life stressors. It has since been adapted to offer a shorter version for use in clinical and research settings.

### Overview of the Religious Coping Scale (RCOPE)

When developing items for the RCOPE, Pargament (1997) used existing religious coping scales and empirical studies to help in this task. Previous clinical experience was also drawn upon, as well as interviews with people who were relying on their religiosity to cope with a variety of major life stressors. Twenty-one subscales were identified with approximately eight items of specific religious coping strategies per subscale, which encompass emotion-focused and problem-focused approaches; passive, active, and interactive strategies; and spiritual, interpersonal, behavioural, and cognitive domains (Pargament et al., 2013). Ten raters (psychology graduates) were then asked to sort scale items into appropriate subscales, and those that were not reliably classified by 80% of the raters were then discarded. The remaining items displayed almost 100% agreement in classification among the raters. This resulted in the final version of the RCOPE consisting of 105 items: five items for each of the 21 subscales.

The final RCOPE is considered to be a multi-functional instrument which reflects the five religious functions as defined by Pargament (1997) in his theory of religious coping (i.e., meaning, intimacy, control, comfort, and life transformation; Pargament et al., 2000). It also reflects the search for spirituality (Pargament et al., 2011). The RCOPE is also considered to be multi-modal, in that items on the scale were selected to represent how people employ their religious coping methods emotionally, relationally, behavioural, and cognitively (Pargament et al., 2000).

It is also multi-valent in nature, with the understanding that coping strategies can be adaptive or maladaptive in dealing with stressful situations (Pargament et al., 2000). As such, items on the RCOPE were selected to reflect both positive and negative religious coping methods. The RCOPE has demonstrated efficacy as a predictive instrument for both psychological and physical adaptation to life stressors; exhibiting superiority over alternative religiosity assessment measures (Pargament et al., 2011).

### Overview of the Brief Religious Coping Scale (Brief RCOPE)

Although the 105-item RCOPE is esteemed for its psychometric merit, its length presents practical challenges for implementation within clinical contexts, particularly where individuals undergoing assessment are expected to complete a comprehensive battery of psychometric evaluations (Fairfax, 2017). The length has resulted in the RCOPE not being used extensively and, as such, findings related to the tool’s effectiveness are limited (Pargament et al., 2011). The Brief RCOPE was developed to address the issue of length (Pargament et al., 1998). It is the most used measure of religious coping and has the potential to be used in both clinical and research settings (Mohammadzadeh & Najafi, 2016).

The development of the Brief RCOPE began at the same time as the full 105-item version, with an abbreviated 21-item scale being tested with a sample of participants who lived near Oklahoma City, following a bomb explosion in 1995. Within the 21-item scale, an exploratory factor analysis revealed two factors which accounted for approximately 33% of the variance; positive and negative coping (Pargament et al., 2011). The authors then began development of a 14-item version of the RCOPE, due to the promising findings.

Based on the two factors identified from the factor analysis, positive and negative religious coping scales (PRC and NRC respectively) were developed using a selection of items from each subset of the RCOPE. The selected items were those that demonstrated large factor loading, and those which contributed to a representation of a variety of coping methods (Pargament et al., 2011). The final version of the Brief RCOPE was therefore divided into two subscales: PRC and NRC methods, each consisting of seven items (Pargament et al., 2011). Table 1, taken from Pargament et al.’s (2011) paper (p. 57), provides an overview of the items in the two scales.

**Table 1:** The brief RCOPE: positive and negative coping subscale items

Item No	*Positive religious coping subscale items*
1	Looked for a stronger connection with God
2	Sought God’s love and care
3	Sought help from God in letting go of my anger
4	Tried to put my plans into action together with God
5	Tried to see how God might be trying to strengthen me in this situation
6	Asked for forgiveness for my sins
7	Focused on religion to stop worrying about my problems
	*Negative religious coping subscale items*
8	Wondered whether God had abandoned me
9	Felt punished by God for my lack of devotion
10	Wondered what I did for God to punish me
11	Questioned God’s love for me
12	Wondered whether my church had abandoned me
13	Decided the devil made this happen
14	Questioned the power of God

Positive religious coping (PRC) strategies include holding a belief in a loving God and believing that difficult situations are opportunities for growth which are sent by God (O’Brien et al., 2019). Individuals who adopt PRC strategies tend to possess a deep sense of spiritual connection with others, have a belief that there is meaning to be found in life, and adopt a compassionate view of the world (Pargament et al., 1998). Positive religious coping strategies have been found to predict increases in positive affect and self-esteem and decreases in depressive symptoms in a sample of 937 African Americans (Park et al., 2018). They have also been found to improve the quality of life of patients with advanced cancer (Tarakeshwar et al., 2006), as well as resulting in a greater sense of meaning in life and less loneliness for adults in Turkey during the Coronavirus disease (COVID-19) crisis (Yıldırım et al., 2021).

Negative religious coping (NRC) strategies refer to ways of dealing with stress that involve difficulties in one’s relationship with God (Pargament et al., 2013). Individuals who adopt NRC strategies tend to experience spiritual tension, instability with others, demonic reappraisals and a fragile view of the universe (Voytenko et al., 2023). This style of coping is assumed to be defined by self-directed religious coping, reappraisals of God’s powers, punitive religious reappraisals, and interpersonal religious disconnect (Pargament et al., 1998).

In the literature, NRC strategies are often referred to as religious struggles, reflecting the struggle in the relationship with God, oneself and with other people (O’ Brien et al., 2019). Negative religious coping has been found to predict: worse overall mental health and life satisfaction in women with breast cancer (Hebert et al., 2009); greater anxiety, worry, and depression on older minority adults (O’Brien et al., 2019); and greater levels of depression and anxiety in Alzheimer caregivers (Gonyea & O’Donnell, 2021). Despite these findings, Pargament (2011) recognised that PRC methods may not always be adaptive, and that NRC methods may not always be maladaptive.

In general, it has been found that people tend to use PRC rather than NRC when dealing with life stressors (Ahles et al., 2016; Hebert et al., 2009; Pargament et al., 1998). A meta-analysis conducted by Ano and Vasconcelles (2005) found that PRC strategies are significantly associated with positive outcomes following stress and fewer experiences of distress, anxiety, and depression. Their findings also suggest that individuals who adopt NRC strategies experienced more distress, anxiety, and depression. Negative religious coping has also been found to predict an increase in posttraumatic symptoms after stressful events in Chilean adults (García et al., 2017) and in individuals who experienced Hurricane Katrina (Henslee et al., 2015). These findings suggest that individuals who demonstrate NRC styles may benefit from receiving help to deal with coping with a crisis (Pargament et al., 1998).

During development, the psychometric properties of the Brief RCOPE were considered, and the internal consistency in the sample of Oklahoma City residents dealing with the aftermath of a bomb explosion was considered high, with Cronbach’s coefficient alpha estimates of 0.90 for the PRC scale and 0.81 for the NRC scale (Pargament et al., 1998).

Following this, the Brief RCOPE scales were used with a hospital sample. Cronbach’s coefficient alpha was estimated at 0.87 for the PRC scale, and 0.69 for the NRC scale, finding them to be internally consistent (Pargament et al., 1998). It was also found that following dealing with a stressor, PRC methods were linked to participants experiencing fewer psychosomatic symptoms and greater spiritual growth. Conversely, poorer quality of life, psychological distress and symptoms, and greater callousness toward other people were correlated with using NRC methods (Pargament et al., 2011).

### Administration of the Brief RCOPE

Users of the 14-item measure are prompted to score each item on a 4-point Likert scale with response options from 0 “not at all” to 3 “a great deal” (Pargament et al., 1998). Higher scores on each subscale indicates greater religious coping of that type (Abu-Raiya et al., 2020). While there is no published manual, guidance provided by Pargament et al. (2011) state that the RCOPE can be adapted to specify different life stressors or to measure coping of general life events. It is also suggested that while the original measure uses the term “God” due to its theistic nature, this can also be changed to meet the needs of the individual completing the measure. For example, “God” could be replaced with “Allah” or “higher power” etc. to account for other religions.

A psychometric critique of the Brief RCOPE was conducted in 2011 by Pargament et al. which concluded that the measure is reliable and valid (i.e., that it has the ability to consistently reproduce results and that it measures what it is proposed to; de Souza et al., 2017), with both the PRC scale and the NRC scale demonstrating good internal consistency across a range of samples. Further research has also found good internal consistency for the two scales, including in a sample of Black Americans living with human immunodeficiency virus (HIV) (Lassiter & Poteat, 2020), in undergraduate students misusing prescription stimulants (Gallucci et al., 2018), and in a sample of young adults who have experienced parental divorce (Milam & Schmidt, 2018).

While these are positive findings, these, and the psychometric properties as reported in Pargament et al.’s (2011) review, were focused mainly on Christians in the United States. Additionally, the RCOPE has been criticised for relying on Christian ideas and core values (Abu-Raiya & Pargament, 2014), and it is recommended that further research should be undertaken to examine the reliability and validity of the Brief RCOPE in non-Christian faiths and in other regions of the world. While religious coping appears to be a universal phenomenon, the particular methods of expression vary between religions (Abu-Raiya & Pargament, 2014). For example, in times of stress, Buddhists may practice mindfulness and compassion (Phillips et al., 2012), Muslims may read the Quran to find comfort, and Jews may wait for the Sabbath (Rosmarin et al., 2009).

Therefore, the aim of this narrative review will be to evaluate the reliability and validity of the Brief RCOPE as applied to cultures and populations outside of the United States. A narrative synthesis of findings of relevant studies is presented below under sub-headings relating to the sub-types of reliability and validity.

## Method

The review took a narrative approach which involved a comprehensive search of peer-reviewed journal articles and book chapters, published in English from the measure’s inception in 2011 to the current year. Sources were identified through electronic databases (PubMed, Web of Science, PsycINFO, and OVID). Keywords were used in each database with relevant truncations. Keywords included "Brief RCOPE," "religious coping," "psychometric properties," "reliability," and "validity".

Inclusion criteria were developed and applied to ensure the relevance of the studies reviewed. Specifically, studies were included if they explored and evaluated the psychometric properties of the Brief RCOPE (e.g., reliability, validity) and involved populations outside the United States, focusing on various cultural and religious contexts. Studies that did not meet the criteria, such as those focusing on other measures of religious coping or those conducted solely within the United States, were excluded. The findings of selected articles were collated and synthesised. Findings related to the internal consistency, test–retest reliability, convergent validity, construct validity, and concurrent validity of the Brief RCOPE. Special attention was given to how the measure performed across different cultural and religious groups, as well as any challenges or limitations identified in its application.

## Results

### Reliability

Reliability refers to the replicability of a test. It is the extent to which a measure is likely to consistently produce similar findings every time it is used (Coaley, 2014). A test with high reliability suggests low error, and conversely a test with low reliability means larger amounts of error (Coaley, 2014). Two types of reliability are considered here: internal reliability, and test–retest reliability.

#### Internal Reliability

Internal reliability is concerned with whether each item on the scale is measuring the same concept (Henson, 2001). Cronbach’s alpha is the most widely used measure of reliability and is used to measure the internal consistency of a scale. It is expressed as a number between 0 and 1, with acceptable values ranging from 0.70 to 0.95 (Tavakol & Dennick, 2011).

Voytenko et al. (2023) used the Brief RCOPE with a sample from four non-Western countries (Columbia, Indonesia, Ukraine, and South Africa) to examine religious coping following interpersonal hurts. Cronbach’s alpha was ≥ 0.85, suggesting good internal reliability. It was also found that PRC and NRC scales associated positively with one another in each country, with the effect size ranging from negligible (r = 0.07) to large (r = 0.56). Furthermore, Yirdong et al. (2023) reported Cronbach’s alphas of α = 0.92 and α = 0.82 for positive and negative religious coping respectively, in their sample of individuals living with HIV in Ghana.

The Brief RCOPE’s internal reliability has further been demonstrated in a range of religions, including Islam (PRC α = 0.89; NRC α = 0.79) (Abu-Raiya & Sulleiman, 2020); Roman Catholicism (PRC scale only α = 0.87; Wnuk, 2021); and Hinduism (α = 0.89) (Grover & Dua, 2019).

Translated versions of the Brief RCOPE have also been investigated for their psychometric properties. For example, internal reliability has been demonstrated in Rezaeipandari et al.’s (2021) study investigating the reliability and validity of the Brief RCOPE among Persian-speaking older adults suffering from multiple chronic diseases. The researchers for this study translated the Brief RCOPE into Persian. The religious denominations represented in this sample include Muslim and Zoroastrian, investigating the PRC and NRC separately***.*** Cronbach’s alpha was estimated for the whole sample and considered to be in the acceptable range (PRC α = 0.74; NRC α = 0.80). Acceptable alpha estimates were also present in the Muslim subgroup (*n* = 200; PRC α = 0.86; NRC α = 0.79) and Zoroastrian subgroup (*n* = 200; PRC α = 0.80; NRC α = 0.73). Similar findings were further demonstrated in Mohammadzadeh and Najadi’s (2016) sample of 339 university students with undefined religious beliefs, using a Persian translated version of the Brief RCOPE. Cronbach’s alpha coefficient was obtained equal to 0.79 and 0.71 for positive and negative religious coping respectively.

Further findings of robust internal reliability have been demonstrated in other Brief RCOPE translations, including in Greek for people with and without long-term conditions (PRC α = 0.91—0.96; NRC α = 0.77 – 0.92; Paika et al., 2017), in Arabic for secondary school students in Iraq (α = 0.70; PRC α = 0.86; NRC α = 0.82; Al-Hadethe et al., 2016), in Portuguese for the Brazilian general public (PRC α = 0.88; NRC α = 0.84; Esperandio et al., 2018), and in Spanish for Mexican Americans with diabetes (PRC α = 0.85; NRC α = 0.86; Martinez & Sousa, 2011).

#### Test–Retest Reliability

Test–retest reliability measures the stability of the measure from the same person across two or more time points (Polit, 2014). This assesses the amount of error due to random fluctuations over time (Coaley, 2014). There is some discrepancy in the literature regarding what is considered to be an acceptable test–retest reliability coefficient, however Polit (2014) suggests that scale developers should aim to achieve test–retest reliability coefficients that exceed 0.80.

In Rezaeipandari et al.’s (2021) study investigating the psychometric properties of the Brief RCOPE in Persian-speaking older adults, an intraclass correlation coefficient was calculated to test the reliability over a two-week time period. Consistency was demonstrated for the PRC (0.89) and the NRC (0.91) subscales. Consistency was also demonstrated across a four-week time period in a sample of Iranian university students (Mohammadzadeh & Najafi, 2016). The test–retest coefficient was obtained for the total scale (0.90), PRC (0.93) and NRC (0.88) (*p* < 0.001).

While there is some available data for the test–retest reliability, this is limited. However, it is likely that coping methods, and indeed stressors, will change over time. Therefore, due to the items measured by the Brief RCOPE being changeable in nature, this type of reliability may not be regularly measured by researchers, due to the expected change in contexts and utility of strategies (Polit, 2014).

### Validity

Broadly speaking, validity refers to a test’s ability to measure what it intends to (Hughes, 2018). Pargament et al.’s (2011) psychometric review demonstrated good validity; however, test validity is improved by ensuring the results of a test apply across a range of populations (Raykov & Marcoulides, 2011). Therefore, this review will now provide an overview of the validity of the Brief RCOPE across a range of populations.

#### Convergent Validity

Convergent validity refers to “the degree to which scores on a studied instrument are related to measures of other constructs that can be expected on theoretical grounds to be close to the one tapped into by this instrument” (Raykov & Marcoulides, 2011, p. 207). A common method of determining convergent validity is through the average variance extracted (AVE) method, recommended by Fornell and Larcker (1981). Average variance extracted is used to measure the amount of variance that is captured by the construct in relation to the amount of variance due to measurement error (dos Santos & Cirillo, 2021). For a measure to establish convergent validity, the AVE should be equal to or greater than 0.50 (Hu & Bentler, 1999). This indicates that 50% or more of the construct’s variance is due to its indicators (Pagán-Torres et al., 2021).

In their validation of the Brief RCOPE on Iranian university students, Mohammadzadeh and Najafi (2016) report that convergent validity coefficients were obtained for the PRC subscale (0.85) and the NRC subscale (0.83). A further attempt to seek validation was conducted by Esperandio et al. (2018) for the Brazilian Brief RCOPE. The researchers used AVE to assess convergent validity; both the PRC and NRC subscales showed an AVE of 0.5, suggesting convergent validity.

Pagán-Torres et al. (2021) also examined the convergent validity of the Brief RCOPE in their sample of Puerto Rican adults. In addition to this, the researchers measured discriminant validity (i.e., the extent to which measures intended to measure distinct constructs do so; Rönkkö & Cho, 2022) by comparing the AVE values for both factors to the average shared variance (ASV) and the maximum shared variance (MSV). For discriminant validity to be determined, the AVE of each factor should be higher than these two measures of variance. The findings here suggest that both the PRC (0.69) and NRC (0.51) factors were higher than the values of the ASV (PRC = 0.03; NRC—0.03) and MSV (PRC = 0.03; NRC—0.03), suggesting discriminant, as well as convergent validity.

#### Construct Validity

Construct validity concerns the degree to which items on an assessment measure what they intend to measure (Stone, 2019). Abu-Raiya et al. (2020) conducted a confirmatory factor analysis to test whether data collected from their sample of 486 Israeli Jews and Muslims fit the two-factor structure of the Brief RCOPE. The model showed a poor fit to the data; therefore, the researchers performed an exploratory factor analysis on the Brief RCOPE’s 14 items. This yielded two factors with eigenvalues greater than 1, accounting for 63% of the variance. On both factors, the item ‘Questioned God’s power’ had factor loadings of less than 0.40 and was therefore eliminated from further analyses.

Similar findings for this item have been demonstrated across the literature in different populations, e.g., in Jews and Muslims (Rezaeipandari et al., 2021), in the Czech Republic (Janu et al., 2019), and across four non-Western countries (Voytenko et al., 2023). It is hypothesised that this may be the case because the meaning of the item may not translate implicitly across cultures. Alternatively, it is hypothesised that in some religions and contexts, questionings God’s power may be more prohibited than in others (Voytenko et al., 2023). Additionally, it is possible that while people may experience their own struggles with their relationship with God, they may not question the nature of God (Janu et al., 2019).

Factor 1’s seven items were examined and were found to be linked to the construct originally labelled PRC (Abu-Raiya et al., 2020). Furthermore, all items linked to Factor 2 were conceptually linked to the construct originally labelled NRC. This demonstrates good construct validity, and similar findings were present when an exploratory factor analysis was performed for Jews and Muslims separately (Abu-Raiya et al., 2020). Further support for construct validity can be found in Voytenko et al.’s (2023) study of four non-Western countries, whereby the two factors were retained.

#### Concurrent Validity

Concurrent validity refers to “the strength of relationship between test scores and criterion measurements made at the time of test administration or shortly thereafter” (Raykov & Marcoulides, 2011, p. 187). Pearson correlation is often used for the examination of concurrent validity. Values less than 0.35 are considered weak or low correlations; values between 0.36 and 0.67 moderate correlations; values between 0.68 and 0.89 are considered high correlations; and, finally, values from 0.90 onwards are considered very high correlations (Taylor, 1990). In Pargament et al.’s (2011) psychometric critique, the Brief RCOPE demonstrated good concurrent validity.

In Mohammadzadeh and Najafi’s (2016) study, concurrent validity referred to how well the Brief RCOPE compared to other psychometric assessments measuring religious coping. They compared the Brief RCOPE, as applied to Iranian university students, to the Islamic Coping Strategies Scale in Stressful Conditions (Ehteshamzadeh, 2009). The results demonstrate that the PRC and NRC scales correlate highly with the Islamic Coping Strategies Scale in Stressful Conditions (PRC = 0.85; NRC = 0.83; p < 0.001), suggesting concurrent validity. Similarly, García et al. (2017) compared the Brief RCOPE to the religiosity subscale of Carver’s Brief-COPE (1997) in their sample of 442 Chilean adults. The PRC and NRC subscales show positive and significant correlations with the religiosity subscale of the Brief RCOPE.

Further support for the concurrent validity of the Brief RCOPE has been demonstrated in a sample of 320 Puerto Rican adults, with the assumption that PRC is mostly associated with measures of positive psychological constructs, and NRC related to signs of poorer mental health (Pargament et al., 2011). Pearson correlation coefficients were conducted on the PRC and NRC subscales. The PRC subscale was correlated with ratings in areas such as participation in private religious practices (r = 0.568, *p* < 0.001), participation in religious practices (r = 0.569, *p* < 0.001), and importance of religion (r = 0.681, *p* < 0.001). The NRC subscale did not correlate with these variables (Pagán-Torres et al., 2021).

Similar findings and support for concurrent validity of the Brief RCOPE were reported in Paika et al.’s (2017) study examining the psychometric properties of the Greek-Orthodox version of the Brief RCOPE. They report that NRC was strongly associated with poorer mental health and greater depressive symptoms, as reported in other studies (Pargament et al., 2011).

### Appropriate Norms

A normative population is required for a tool to be considered useful (Lenhard et al., 2019). The norm allows the score to be interpreted at a group or an individual level, without which the score would be meaningless (Peña et al., 2006). Pargament et al. (2011) reported that mean scores for PRC and NRC can range from 7 (minimum) to 28 (maximum) when a four-point (1-to-4) Likert style scale is used. In their review of studies, mean scores ranged from 17 to 21 for PRC, suggesting that respondents tend to favour “somewhat” or “a great deal” answers on the scale. The mean scores for the NRC subscale ranged from 8 to 14, suggesting that respondents tend to favour “not at all” or “somewhat” for the NRC items. As stated earlier, Pargament et al.’s (2011) review was conducted mainly on Christians in the United States. There is currently no available normative data available for the Brief RCOPE in non-Western cultures.

### Strengths and Limitations of the Review

This review has placed a focus on the use of the Brief RCOPE with a range of populations across multiple countries and cultures. In so doing, it provides insight into the use of the measure with diverse populations; insight which is essential for practitioners and researchers using this measure outside of the population for which it was first developed. To the authors’ knowledge, it is the first review to undertake this task.

However, the review only includes papers which were published in the English language and, as such, there may be research which was not included leading to potential bias. This was unavoidable due to time and resources available to the authors. In addition, it only included peer review papers, and the challenges of publishing literature in international journals may have led to further bias in the findings reported in this article.

Furthermore, the narrative approach taken may introduce subjectivity in the selection of articles for inclusion, potentially leading to bias in the reviewed literature. Lastly, synthesising findings from such diverse sources, as presented above, can be challenging and may lead to oversimplification or the overlooking of important nuances.

## Conclusion

The Brief RCOPE remains the most used measure of religious coping and has the potential to be used across different religions, cultures, and in relation to a variety of different life stressors. Overall, good reliability has been demonstrated across a number of countries, and across a variety of different religions. The measure has also demonstrated good convergent, construct and concurrent validity in diverse cultures. The translated versions of the Brief RCOPE have demonstrated similar results to those obtained for the original measure, suggesting that these tools can be used to assess religious coping in people exposed to stressful events in these cultures (García et al., 2017). Unfortunately, normative data for a non-Western population has not been specified, resulting in difficulties comparing individual scores to the wider population.

Pargament et al. (1998) suggests that the Brief RCOPE has practical applicability for mental health, religious, or health professionals for informing assessment and intervention. They report that religiously oriented interventions to support people to cope with major life stress could be based on the positive patterns of religious coping. Furthermore, individuals who demonstrate negative religious coping styles may benefit from help to deal with coping with a crisis (Pargament, 1998). Ano and Vasconcelles (2005) reason that it is important to explore an individual’s religion coping strategies, as it allows practitioners to determine whether religion serves as an adaptive or a maladaptive method of coping and may help practitioners identify potential warning signs that there may be issues related to an individual’s religious coping. The findings from this review suggest that the Brief RCOPE can also be used in a range of diverse religions and cultures.

However, the majority of studies investigating the psychometric properties of a translated version of the Brief RCOPE found issues with item 14 (“Questioned the power of God”) and as a result were required to remove it from their analysis. Pagán-Torres et al. (2021) report that had they not removed it from their investigation, the alpha coefficient would be reduced, and it would lose its convergent reliability. Therefore, users of the original Brief RCOPE with diverse populations should be mindful of the implications of including item 14 in their assessment battery.

In addition, more research is required to test the applicability of the Brief RCOPE on a wider range of populations, for example, across other cultures/religions and with vulnerable populations such as those with mental health issues, prisoners, and victims of war and crime who are likely to have different life experiences and cognitions than others in the general population.
